# Epidemiology of *Streptococcus pneumonia*e Serogroup 6 Isolates from IPD in Children and Adults in Germany

**DOI:** 10.1371/journal.pone.0060848

**Published:** 2013-04-09

**Authors:** Mark van der Linden, Nadine Winkel, Sharon Küntzel, Aron Farkas, Stephanie Russo Perniciaro, Ralf René Reinert, Matthias Imöhl

**Affiliations:** National Reference Center for Streptococci, Department of Medical Microbiology, University Hospital (RWTH), Aachen, Germany; Centers for Disease Control & Prevention, United States of America

## Abstract

This study presents serogroup 6 isolates from invasive pneumococcal disease (IPD) before and after the recommendation for childhood pneumococcal conjugate vaccination in Germany (July 2006). A total of 19,299 (children: 3508, adults: 15,791) isolates were serotyped. Serogroup 6 isolates accounted for 9.5% (children) and 6.7% (adults), respectively. 548 isolates had serotype 6A, 558 had serotype 6B, 285 had serotype 6C, and 4 had serotype 6D. Among children, serotype 6B was most prevalent (7.5% of isolates) before vaccination, followed by 6A and 6C. After the 7-valent pneumococcal conjugate vaccine (PCV7), the prevalence of serotype 6B significantly decreased (p = 0.040), a pattern which continued in the higher-valent PCV period (PCV10, PCV13). Serotype 6A prevalence showed a slight increase directly after the start of PCV7 vaccination, followed by a decrease which continued throughout the PCV10/13 period. Serotype 6C prevalence remained low. Serotype 6D was not found among IPD isolates from children. Among adults, prevalence of both 6A and 6B decreased, with 6B reaching statistical significance (p = 0.045) and 6A showing a small increase in 2011–2012. Serotype 6C prevalence was 1.5% or lower before vaccination, but increased post-vaccination to 3.6% in 2011/12 (p = 0.031). Four serotype 6D isolates were found post-PCV7 childhood vaccination, and two post-PCV10/13. Antibiotic resistance was found mainly in serotype 6B; serotype 6A showed lower resistance rates. Serotype 6C isolates only showed resistance among adults; serotype 6D isolates showed no resistance. Multilocus sequence typing showed that sequence type (ST) 1692 was the most prevalent serotype 6C clone. Thirty-two other STs were found among serotype 6C isolates, of which 12 have not been previously reported. The four serotype 6D isolates had ST 948, ST 2185 and two new STs: 8422 and 8442. Two serogroup 6 isolates could not be assigned to a serotype, but had STs common to serogroup 6.

## Introduction

*Streptococcus pneumoniae* remains a major cause of infectious disease globally, especially in children. Invasive pneumococcal disease (IPD) causes an estimated 0.5 million deaths among children worldwide [1]. The most important virulence factor of *S. pneumoniae* is the polysaccharide capsule. Currently, 94 capsular types, or serotypes, have been described.

Before the introduction of pneumococcal conjugate vaccination, serogroup 6 was one of the most common causes of IPD among children and adults. Originally, serogroup 6 comprised two serotypes, 6A and 6B. Using monoclonal antibodies, a new serotype, serotype 6C, could be identified among isolates previously typed as 6A. In serotype 6C, the capsular locus of the *wciN_α_* gene has been replaced through homologous recombination by the *wciN_β_* gene [2]. By cloning the *wciN_β_* gene into a 6B strain, the possibility of yet another subtype, serotype 6D, was proven in a lab environment [3]. Shortly after that, serotype 6D was found in clinical isolates [4]–[6]. Currently, serogroup 6 contains four serotypes, with serotype 6A having the *wciP_α_* gene and the *wciN_α_* gene, 6B (*wciP_β_*, *wciN_α_*), 6C (*wciP_α_*, *wciN_β_*) and 6D (*wciP_β_*, *wciN_β_*) [7].

Some serotype 6A and 6B isolates contain a 300 bp long insertion between the *wciO* and the *wciN* gene. Based on the presence of this INDEL sequence, the gene loci are divided into class I (with INDEL) and class II (without INDEL) sequences. The majority of 6A and 6B isolates do not carry an INDEL and belong to class I [7].

Since 2001, a pneumococcal conjugate vaccine (PCV7) has been available, covering the seven serotypes most prevalent in invasive pneumococcal disease (4, 6B, 9V, 14, 18C, 19F and 23F). After introduction of this vaccine in the US, a strong reduction of the incidence of vaccine-type IPD has been observed [8]. PCV7 includes serotype 6B. It was suggested, on the basis of antibody ELISA and opsonophagocytosis tests, that PCV7 elicits some level of cross-protection to serotype 6A [9]. In a study from the US, it was shown that this was indeed the case, since the incidence of serotype 6A decreased after introduction of the vaccine [10], [11]. However, PCV7 does not provide cross-protection to serotype 6C, and an increase in serotype 6C was observed in the US after introduction of PCV7 [11].

In 2009, higher-valent pneumococcal conjugate vaccines were licensed in Germany. A 10-valent vaccine (PCV10) contains the PCV7 serotypes, plus serotypes 1, 5 and 7F, and a 13-valent vaccine (PCV13) is comprised of serotypes 3, 6A and 19A in addition to the PCV10 serotypes. For PCV13, a cross-protection from serotype 6A antibodies for serotype 6C was speculated, because PCV13 immune sera showed strong opsonophagocytic responses to serotype 6C [12]. In a study on children with acute otitis media in France, Cohen et al. showed that in PCV13-vaccinated children, carriage of serotype 6C was significantly reduced as compared to PCV7-vaccinated children [13].

In Germany, a general recommendation for pneumococcal conjugate vaccination for all children under two years of age was issued in July 2006. Initially, vaccination was performed with PCV7. Beginning in 2009, with the licensing of higher-valent vaccine formulations, PCV10 was used in vaccinations from April of that year, and PCV13, which replaced PCV7, was introduced in December 2009. The aim of this study was to determine the effects of this vaccination recommendation on invasive pneumococcal disease caused by serogroup 6. Furthermore, we aimed to determine the presence of serotypes 6C and 6D among invasive isolates from both adults and children, and to verify their role in the epidemiology of serogroup 6 IPD after the start of vaccination.

## Materials and Methods

### Study Material

Serogroup 6 isolates were obtained from ongoing surveillance studies on IPD in children in Germany under 16 years of age from July 1997 to June 2012 [14] and from adults 16 years and older from July 1992 to June 2012 [15]. *S. pneumoniae* samples isolated from blood, CSF or other normally sterile body sites were included in the study. Cases of IPD were grouped per pneumococcal season (from July in one year til June in the following year), since infections tend to cluster around the winter months.

### Serotyping

All pneumococcal isolates were serotyped using the Neufeld’s Quellung reaction with group and factor sera provided by the Statens Serum Institut, Copenhagen, Denmark. All serotype 6A and 6B isolates were revived from frozen stocks and retested for serotypes 6C and 6D. Initially, 6A isolates were tested for 6C using the PCR method described by Park [2]. As new factor sera 6b, 6c and 6d became available from the Statens Serum Institut, these were used for analysis, with serotype 6A testing positive for factor serum 6b, serotype 6B testing positive for factor serum 6c and serotype 6C testing positive for factor serum 6d. Serotype 6B isolates were tested with sera only. Isolates positive for 6D (reacting with factor sera 6b and 6d) were verified with several PCRs using primers described by Jin *et al*. [16]. Using a random number generator in MS-Excel, a sampling of serotype 6A and 6B isolates were selected and tested for the presence of the INDEL sequence between the *wciN* and *wciO* genes [7], [17] using the same primers as described above [2].

### Susceptibility Testing

All isolates were tested for antibiotic minimal inhibitory concentrations (MIC) using the broth microdilution method as recommended by the Clinical and Laboratory Standards Institute (CLSI) [18]. The microtiter plates (Sensititre NLMMCS10, TREK Diagnostic Systems Ltd., East Grinstead, UK) contained penicillin G, clarithromycin (erythromycin before 2004 and after 2011), clindamycin and tetracycline with cation adjusted Mueller-Hinton broth (Oxoid, Wesel, Germany) and 5% lysed horse blood. The final inoculum was 5×10^5^ CFU/ml. Incubation was at 37°C for 24 h in ambient air. *S. pneumoniae* ATCC 49619 was used as a control strain. For interpretation, the current CLSI criteria were applied [18]. For penicillin susceptibility, the ‘oral’ breakpoints were used (≤0.06 µg/ml, 0.12–1 µg/ml, ≥2 µg/ml), since they provide better insight into resistance development over time.

### Multilocus Sequence Typing

Multilocus sequence typing of selected serogroup 6 isolates was performed as described previously [19]. Briefly, internal fragments of the *aroE, gdh, gki, recP, spi, xpt* and *ddl* genes were amplified by PCR from chromosomal DNA with the described primer pairs. A special allelic profile was provided by the alleles at each of the seven loci and their sequence type (ST) was defined. The allelic profiles were compared with each other and with other isolates in the pneumococcal MLST database using software available at www.mlst.net. Clusters of related STs were grouped into clonal complexes (CCs) using the program eBURST (www.mlst.net).

### Statistical Methods

Statistics were performed using the 'Analysis Toolpak' in MS-Excel. To compare the serotype prevalences before and after the introduction of PCV7 vaccination, the two-sample t-Test assuming equal variances was used. Results were considered significant when p<0.05.

## Results

A total of 3508 isolates from invasive pneumococcal disease (IPD) in children (collection period: July 1997 til June 2012) and 16,109 from IPD in adults (collection period: July 1992 til June 2012) were sent to the German National Reference Center for Streptococci for typing.

Among IPD in children, isolate numbers varied from 152 in 1998/99 to 295 in 2006/07. The average number of isolates per pneumococcal season was 234. All 3508 isolates were serotyped. Among adults, isolate numbers varied from 172 in 1996/1997 to 2301 in 2010/11 (average: 805). In the years 1992–1996, the numbers of collected isolates were considerably lower. In January 2007, an enhanced, web-based reporting system (Pneumoweb) was installed, which increased the number of reported isolates considerably (**Table 1**). Previously, we have reported that in the years before 2004, not all adult IPD isolates had been serotyped [20]. We have now been able to type most of these not-typed isolates, resulting in 15,791 serotyped isolates and an overall serotyping rate of 98% (80–100%).

**Table 1:** Isolates from IPD in German children and adults from July 1997 to June 2012 and serogroup 6 isolates included in this study.

	children								adults							
pneumococcal season	all cases	serotyped	% serotyped	6A (%)	6B (%)	6C (%)	6D (%)	SG6(%)	all cases	serotyped	% serotyped	6A (%)	6B (%)	6C (%)	6D (%)	SG6(%)
**pre-vaccination**																
**1992–1993**									404	323	80.0	7 (2.2)	11 (3.4)	2 (0.6)	0 (0.0)	20 (6.2)
**1993–1994**									299	270	90.3	9 (3.3)	13 (4.8)	3(1.1)	0 (0.0)	25 (9.3)
**1994–1995**									317	267	84.2	9 (3.4)	10 (3.7)	1 (0.4)	0 (0.0)	20 (7.5)
**1995–1996**									270	225	83.3	8 (3.6)	9 (4.0)	3 (1.3)	0 (0.0)	20 (8.9)
**1996–1997**									172	142	82.6	4 (2.8)	6 (4.2)	2 (1.4)	0 (0.0)	12 (8.5)
**1997–1998**	167	167	100.0	5 (3.0)	12 (7.2)	0 (0.0)	0 (0.0)	17 (10.2)	214	182	85.0	8 (4.4)	6 (3.3)	1 (0.5)	0 (0.0)	15 (8.2)
**1998–1999**	152	152	100.0	5 (3.3)	6 (3.9)	1 (0.7)	0 (0.0)	12 (7.9)	240	213	88.8	13 (6.1)	5 (2.3)	0 (0.0)	0 (0.0)	18 (8.5)
**1999–2000**	190	190	100.0	8 (4.2)	13 (6.8)	0 (0.0)	0 (0.0)	21 (11.1)	202	201	99.5	8 (4.0)	2 (1.0)	3 (1.5)	0 (0.0)	13 (6.5)
**2000–2001**	238	238	100.0	11 (4.6)	13 (5.5)	1 (0.4)	0 (0.0)	25 (10.5)	281	274	97.5	11 (4.0)	21 (7.7)	3 (1.1)	0 (0.0)	35 (12.8)
**2001–2002**	241	241	100.0	8 (3.3)	29 (8.3)	2 (0.8)	0 (0.0)	30 (12.4)	423	420	99.3	8 (1.9)	13 (3.1)	2 (0.5)	0 (0.0)	23 (5.5)
**2002–2003**	239	239	100.0	9 (3.8)	21 (8.8)	3 (1.3)	0 (0.0)	33 (13.8)	422	419	99.3	14 (3.3)	21 (5.0)	1 (0.2)	0 (0.0)	36 (8.6)
**2003–2004**	272	272	100.0	10 (3.7)	17 (6.3)	1 (0.4)	0 (0.0)	28 (10.3)	406	403	99.3	10 (2.5)	18 (4.5)	3 (0.7)	0 (0.0)	31 (7.7)
**2004–2005**	286	286	100.0	7 2.4)	31 (10.8)	0 (0.0)	0 (0.0)	38 (13.3)	572	568	99.3	17 (3.0)	19 (3.3)	2 (0.4)	0 (0.0)	38 (6.7)
**2005–2006**	291	291	100.0	9 (3.1)	23 (7.9)	1 (0.3)	0 (0.0)	33 (11.3)	553	553	100.0	12 (2.2)	26 (4.7)	3 (0.5)	0 (0.0)	41 (7.4)
**post-PCV7-vaccination**																
**2006–2007**	295	295	100.0	10 (3.4)	19 (6.4)	0 (0.0)	0 (0.0)	29 (9.8)	1229	1227	99.8	51 (4.2)	36 (2.9)	7 (0.6)	0 (0.0)	94 (7.7)
**2007–2008**	241	241	100.0	10 (4.1)	16 (6.6)	1 (0.4)	0 (0.0)	27 11.2)	1757	1757	100.0	63 (3.6)	45 (2.6)	17 (1.0)	1 (0.1)	126 (7.2)
**2008–2009**	249	249	100.0	12 (4.8)	6 (2.4)	0 (0.0)	0 (0.0)	18 (7.2)	1993	1992	99.9	53 (2.7)	39 (2.0)	39 (2.0)	1 (0.1)	132 (6.6)
**2009–2010**	233	233	100.0	6 (2.6)	3 (1.3)	2 (0.9)	0 (0.0)	11 (4.7)	1996	1996	100.0	58 (2.9)	26 (1.3)	43 (2.2)	0 (0.0)	127 (6.4)
**post-higher-valent-vaccination**																
**2010–2011**	234	234	100.0	1 (0.4)	3 (1.3)	1 (0.4)	0 (0.0)	5 (2.1)	2301	2301	100.0	31 (1.3)	15 (0.7)	60 (2.6)	2 (0.1)	108 (4.7)
**2011–2012**	180	180	100.0	5 (2.8)	1 (0.6)	2 (1.1)	0 (0.0)	8 (4.4)	2058	2058	100.0	38 (1.8)	13 (0.6)	75 (3.6)	0 (0.0)	126 (6.1)
**overall total**	**3508**	**3508**	**100.0**	**116 (3.3)**	**204 (5.8)**	**15 (0.4)**	**0 (0.0)**	**335 (9.5)**	**16109**	**15791**	**98.0**	**432 (2.7)**	**354 (2.2)**	**270 (1.7)**	**4 (0.0)**	**1060 (6.7)**

* Comparison of pre-vaccination (1997–2006, 1992–2006 respectively) and post-vaccination (2006–2012) prevalences showed a statistically significant difference. Serotype 6B children: p = 0.040, serotype 6B adults: p = 0.045, serotype 6C adults: p = 0.031).

A total of 834 serotype 6A isolates were retested for serotype 6C using either PCR or factor serum 6d. 546 were retyped as 6A; 285 as 6C. Two 6A isolates could not be recovered, and one 6A isolate yielded ambiguous results. 563 6B isolates were retested, resulting in 548 6B and four 6D isolates. Ten 6B isolates could not be recovered for retesting, and for one 6B isolate, subtyping was ambiguous. In further calculations, the non-recoverable isolates were treated as having their original serotypes (6A and 6B). The two ambiguous isolates were taken out of the serotype distribution analysis.

A subset of 95 serotype 6B and 47 serotype 6A isolates was tested for the presence of the INDEL described by Mavroidi [7] and Jin [16]. The INDEL was found in 44 (46%) of the serotype 6B isolates but in none of the serotype 6A isolates.

**Figure 1:**
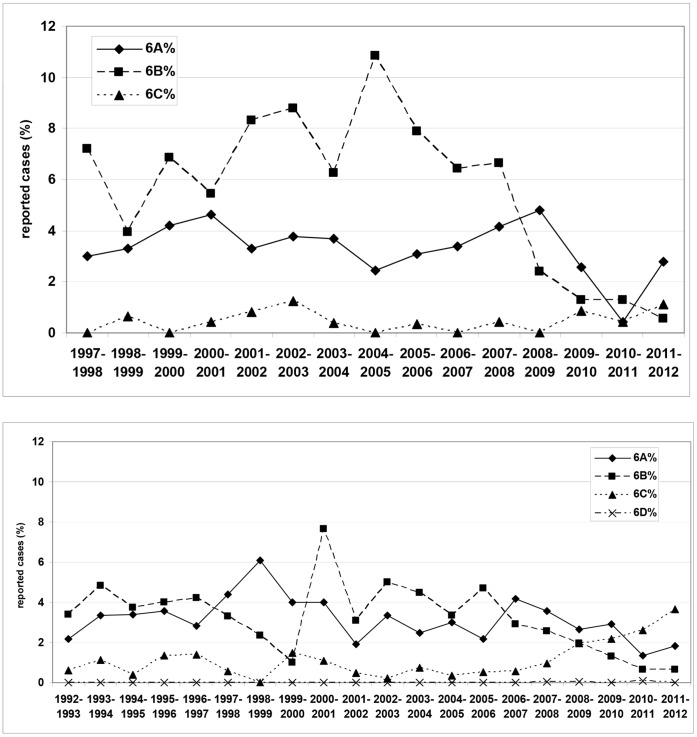
Prevalence of serogroup 6 serotypes over time among isolates from IPD among German children (top) and adults (bottom).

Among children, serotype 6B was most prevalent before the introduction of pneumococcal conjugate vaccination, followed by serotype 6A. After the start of PCV7 vaccination, both the percentage as well as the absolute numbers of isolates of serotype 6B strongly decreased. The decrease continued in the higher-valent PCV period. When comparing the pre-vaccination period (1997–2006) with the post vaccination period (2006–2012), the decrease reached statistical significance (p = 0.040). Serotype 6A prevalence showed a slight increase in the first two years after the start of PCV7 vaccination, followed by a decrease which started in 2009/2010 and continued in the PCV10/13 period. Absolute numbers varied between 1 and 12 isolates per season over the years, decreasing from 12 in 2008/09 to 1 in 2010/11. An increase was observed in 2011–2012, with 5 cases. Three cases were from non-vaccinated children, one from a 4-month-old child that got infected 4 days after the first PCV10 vaccination, and for one case vaccination data could not be obtained. Serotype 6C prevalence remained extremely low, with isolate numbers varying from 0 to 3 per season. Serotype 6D was not found among isolates from children (**Table 1** **,** **Figure 1**).

Among adults, serotypes 6A and 6B showed similar prevalences in the earlier years of the study. 6B showed a diminishing trend until 1999–2000, with a strong increase in the following season. After the start of childhood vaccination, prevalences of both 6A and 6B decreased, with 6A showing a small increase in 2011–2012. The decrease in serotype 6B was statistically significant, with p = 0.045. Serotype 6C prevalence fluctuated between 0.0 and 1.5% before vaccination, with case numbers varying between 0 and 3 per season, but after vaccination increased to 3.6% (75 cases) in 2011/12. The increase was statistically significant (p = 0.031). Serotype 6D was found in four cases, the oldest one dating from July 2007 (**Table 1** **,** **Figure 1**). Isolates were from blood (3) and pleural fluid (1). Data on diagnoses were not available. Patient ages were 48, 61, 75 and 76 years.

**Figure 2:**
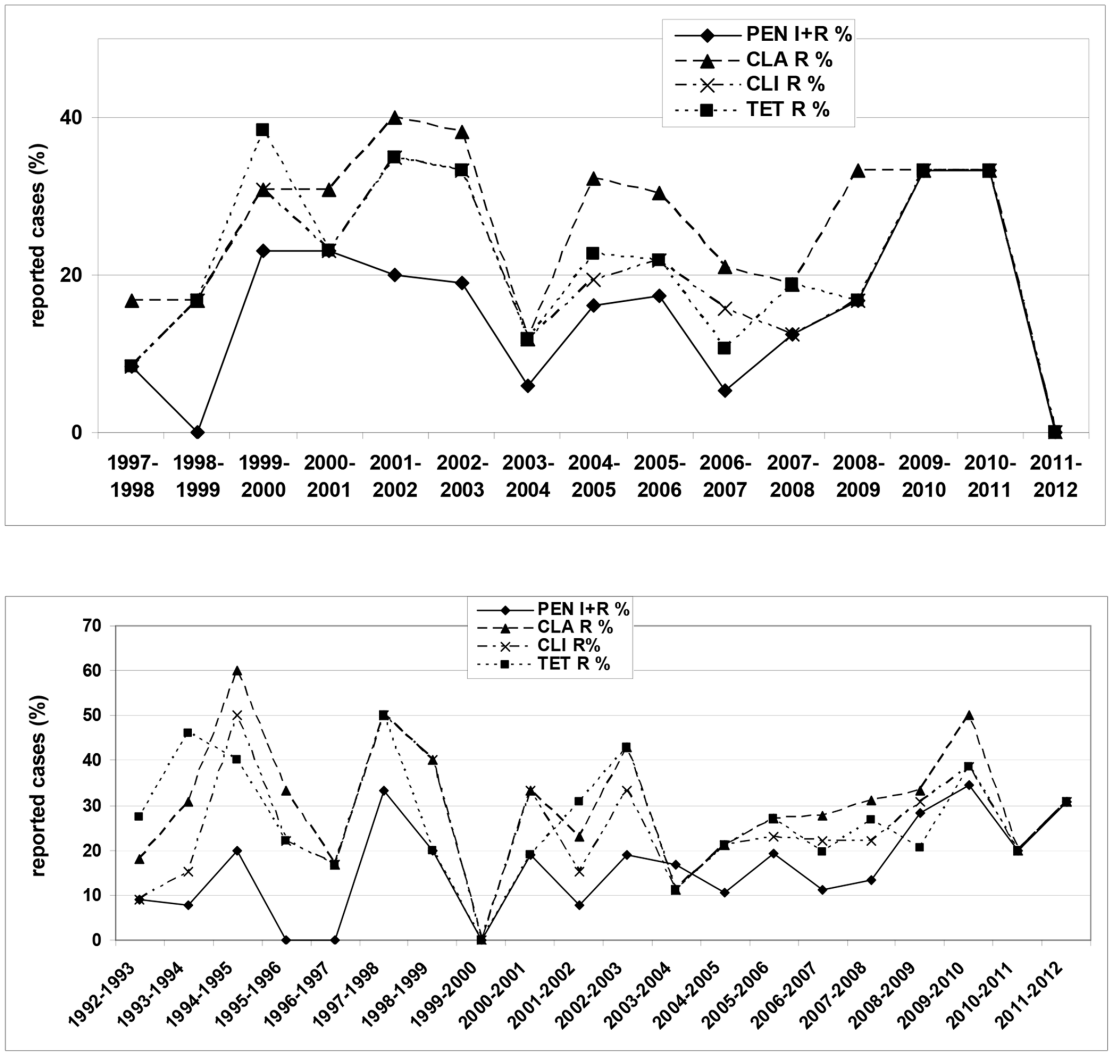
Percentage of resistant serotype 6B isolates from IPD among German children (top) and adults (bottom). PEN: penicillin (breakpoints S, I, R (µg/ml ): ≤0.06, 0.12–1, ≥2), CLA: clarithromycin (breakpoints S, I, R (µg/ml ): ≤0.25, 0.5, ≥1), CLI: clindamycin(breakpoints S, I, R (µg/ml ): ≤0.25, 0.5, ≥1), TET: tetracycline (breakpoints S, I, R (µg/ml ): ≤2, 4, ≥8).

Minimal inhibitory concentration analysis showed that for both children and adults the burden of resistance was found among the serotype 6B isolates, with a penicillin non-susceptibility of 15.2% among children (adults 17.8%), macrolides 27.9% (31.1%), clindamycin 21.6% (25.7%), and tetracycline 22.5% (26.6%). Resistance levels among serotype 6A isolates were considerably lower for isolates from both children and adults (penicillin 4.3% (4.9%), macrolides 8.6% (10.2%), clindamycin 1.7% (3.0%), and tetracycline 4.3% (5.8%)). Resistance levels among serotype 6C from adults (penicillin 6.3%, macrolides 6.3%, clindamycin 3.7%, and tetracycline 4.8%) were comparable to the levels found for serotype 6A, although, following the vaccination period, there were statistically-significant changes in the rate of clarithromycin- and clindamycin- resistant in 6C isolates (p = 0.00026 and p = 0.0025, respectively). The larger part of the resistant 6B isolates were multi drug resistant (MDR; children 64.4%, adults 65.6%), i.e. resistant to more than two different classes of antibiotics. For 6A (children 15.4%, adults 12.5%) and 6C (adults 33.3%) isolates, these percentages were lower. Among serotype 6C isolates from children and 6D isolates from adults, no antibiotic resistant isolates were found (**Table 2** **,** **Figure 2**).

**Table 2:** Antibiotic resistance levels of serogroup 6 isolates from IPD among German children and adults.

serotype	n =	PEN I+R (%)	CLA R (%)	CLI R(%)	TET R (%)	MDR(%)
**children**						
**6A**	116	5 (4.3)	10 (8.6)	2 (1.7)	5 (4.3)	2 (1.7)
**6B**	204	31 (15.2)	57 (27.9)	44 (21.6)	46 (22.5)	42 (20.6)
**6C**	15	0 (0.0)	0 (0.0)	0 (0.0)	0 (0.0)	0 (0.0)
**SG6**	335	38 (11.3)	67 (20.0)	46 (13.7)	51 (15.2)	44 (13.1%)
**all serotypes**	3508	257 (7.3)	755 (21.5)	217 (6.2)	280 (8.0)	190 (5.4%)
**adults**						
**6A**	432	21 (4.9)	44 (10.2)	13 (3.0)	25 (5.8)	10 (2.3)
**6B**	354	63 (17.8)	110 (31.1)	91 (25.7)	94 (26.6)	87 (24.6)
**6C**	270	17 (6.3)	17 (6.3)	10 (3.7)	13 (4.8)	10 (3.7)
**6D**	4	0 (0.0)	0 (0.0)	0 (0.0)	0 (0.0)	0 (0.0)
**SG6**	1062	101 (9.5)	171 (16.1)	113 (10.6)	127 (12.0)	107 (10.1%)
**all serotypes**	15791	953 (6.0)	1857 (11.8)	784 (5.0)	1098 (7.0)	714 (4.5%)

PEN: penicillin (breakpoints S, I, R (µg/ml ): ≤0.06, 0.12–1, ≥2), CLA: clarithromycin (breakpoints S, I, R (µg/ml ): ≤0.25, 0.5, ≥1), CLI: clindamycin (breakpoints S, I, R (µg/ml ): ≤0.25, 0.5, ≥1), TET: tetracycline (breakpoints S, I, R (µg/ml ): ≤2, 4, ≥8), MDR: multi drug resistant (resistant to ≥3 classes of antibiotics).

The two serogroup 6 isolates that could not be assigned to a serotype using factor sera were studied in more detail. The 6B/6D isolate reacted positively to factor sera 6c and 6d, which serologically identified it as serotype 6D. PCR analysis was positive for the *wciPβ* and *wciNα*, characteristic for serotype 6B. An INDEL sequence could not be detected. The sequence type of the isolate was 176, an ST associated with 6A, 6B and 6C in the MLST database (www.mlst.net). The 6A/6C isolate was positive for factor sera 6b and 6d, which makes exact serological typing impossible, since 6b is specific for serotype 6A and 6d is specific for serotype 6C. PCR analysis was positive for *wciPα* and *wciNα*, which is characteristic for serotype 6A. An INDEL sequence was not detected. The isolate had ST 681, associated with serotypes 6A and 6C, but not with 6B in the database (**Table 3**).

**Table 3:** Characteristic of two serogroup 6 isolates which could not be unambiguously assigned to any of the four serotypes.

Isolate	Serotype	MLST	aroE	gdh	gki	recP	spi	xpt	ddl	Factor sera	*wciP*	*wciN*	INDEL
PS 6657	6B or 6D	176	7	13	8	6	10	6	14	6c +, 6d +	*wciPβ*	*wciNα*	no
PS 16864	6A or 6C	681	2	5	9	1	6	19	14	6b +, 6d +	*wciPα*	*wciNα*	no

Apart from the two isolates that gave ambiguous serotyping results, MLST was performed on 172 other serogroup 6 isolates (6A: 30, 6B: 78, 6C: 60, 6D: 4). This included all serotype 6C isolates from children, and 45 out of 270 6C isolates from adults, and all 4 6D isolates from adults. ST 1692 was the most prevalent serotype 6C clone among both children (n = 9) and adults (n = 11). For serotype 6C isolates, 32 other STs were found, of which 12 had not been reported before for serotype 6C (www.mlst.net). The four serotype 6D isolates had ST 948, ST 2185 and two new STs: 8422 and 8442 (**Table 4**). Three 6B isolates with an INDEL for which MLST was performed showed ST 273 (n = 2) and ST 176. Both STs also appear in serogroup 6 isolates without an INDEL.

**Table 4:** MLSTs of serotype 6C isolates from IPD among German children and adults.

Serotype	n =	MLST	aroE	gdh	gki	recP	spi	xpt	ddl
**children**									
6C	9	1692	1	5	7	12	17	158	14
6C	2	600	5	10	9	43	13	1	14
6C	1	8632	5	10	9	43	13	506	14
6C	1	176	7	13	8	6	10	6	14
6C	1	681	2	5	9	1	6	19	14
6C	1	8633	2	10	378	18	27	1	31
**adults**									
6C	11	1692	1	5	7	12	17	158	14
6C	2	473	7	25	4	4	15	20	28
6C	2	1390	10	13	1	43	98	1	20
6C	2	8613	10	25	12	1	15	20	28
6C	2	8633	2	10	378	18	27	1	31
6C	1	176	7	13	8	6	10	6	14
6C	1	207	10	8	30	5	6	1	9
6C	1	224	10	25	8	6	25	6	8
6C	1	367	2	22	4	18	27	4	8
6C	1	395	1	5	7	12	17	1	14
6C	1	460	5	7	4	10	10	1	27
6C	1	600	5	10	9	43	13	1	14
6C	1	667	8	13	14	4	14	4	14
6C	1	681	2	5	9	1	6	19	14
6C	1	1014	2	10	1	43	6	31	6
6C	1	1092	2	13	2	1	6	19	14
6C	1	1150	7	25	8	6	25	6	8
6C	1	1640	13	1	1	2	6	1	18
6C	1	1714	1	5	7	12	17	148	14
6C	1	2185	1	10	9	43	5	1	6
6C	1	2667	7	25	8	16	25	6	8
6C	1	3127	1	25	4	4	15	28	28
6C	1	4269	7	5	4	5	6	1	18
6C	1	8396	7	5	4	6	25	6	8
6C	1	8611	10	13	9	43	6	19	14
6C	1	8612	7	5	8	6	6	20	8
6C	1	8614	1	25	8	16	25	6	8
6C	1	8630	2	379	2	17	6	22	14
6C	1	8615	32	28	7	1	15	52	14
6C	1	8631	32	28	377	1	15	52	14
6C	1	8628	2	378	9	1	6	19	14

**Figure 3:**
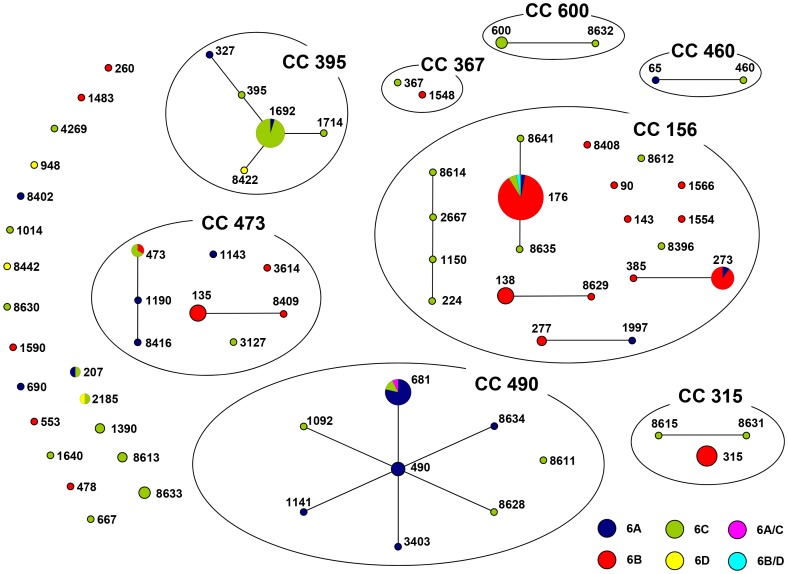
eBURST snapshot of serogroup 6 isolates from IPD in children and adults in Germany. Serotypes are color coded. Circles are proportional to the amount of isolates.

eBURST analysis on all 174 serogroup 6 isolates revealed three clonal complexes (CCs), in which three serogroup 6 serotypes were represented, and 6 CCs with two serogroup 6 serotypes. In 15 CCs only one representative was found; four STs were singletons (**Fig. 3**).

## Discussion

A general recommendation for childhood pneumococcal conjugate vaccination was issued in Germany in July 2006. In this paper we evaluated the effects of this vaccination recommendation on invasive pneumococcal disease caused by serogroup 6. We also determined the prevalence of the two new serotypes, 6C and 6D, among isolates from IPD in Germany.

Until the introduction of childhood conjugate vaccination in 2006, serogroup 6 isolates have always played an important role in IPD among children in Germany, with percentages of serotype 6B varying between 3.9 and 10.8%, and serotype 6A between 2.4 and 4.6% of all isolates.

The amount of serotype 6C isolates has always been very low (1–3 isolates per season). After the introduction of childhood conjugate vaccination with PCV7, a decrease in serotype 6B, starting in 2008–2009, was observed. A year later, in 2009–2010, serotype 6A also started to decrease, most probably due to cross-protection from serotype 6B antibodies [9]–[11]. The sudden increase in serotype 6A in 2011–2012, was due to 5 cases, of which 3 were unvaccinated children. One child was vaccinated with one primary dose only 4 days before infection, which is too short a period to develop immunological protection. For one child, vaccination data could not be obtained. The sudden increase in serotype 6A therefore emphasizes the importance of timely vaccination. Levels of serotype 6C remained unchanged and very low, and do not allow for conclusions on cross protection from any conjugate vaccine.

Among isolates from IPD in adults, serotypes 6A (1.9–6.1%) and 6B (1.0–7.7%) have played an equally important role. After the start of childhood PCV7-vaccination, a reduction in serotype 6B IPD cases was also observed in adults. The reduction started in 2007–2008, continued until 2011–2012, and is most likely due to herd protection effects, which are apparently of similar strength for all three PCVs. After PCV7 childhood vaccination, serotype 6A levels started to decrease in 2007–2008 and leveled off in 2009–2010. The introduction of higher-valent vaccination in children caused another strong decrease in 2010–2011, which again leveled off in 2011–2012. This seems to indicate a stronger protective effect from the higher-valent PCVs, and seems to be in line with the fact that about 80% of all children in Germany are vaccinated with PCV13, which contains both serotype 6A and 6B.

The levels of serotype 6C have been low in the pre-vaccination period (0.0–1.5%), but gradually increased post-vaccination, up to a level of 3.6% in 2011–2012. The levels indicate no herd protection for serotype 6C from PCV7 childhood vaccination. A possible herd protection effect from higher-valent vaccination has also not been observed to date. The remaining level of serotype 6A and the increase in serotype 6C among adults seem to indicate that an eradication of these serotypes is not possible through herd protection alone, and might need adult vaccination.

An increase of serotype 6C isolates, first among adults and later in children, has been reported in Spain [21]. In the US, an increase in 6C was observed after the introduction of vaccination among both children and adults, even though levels of 6C remained low [11]. In Germany, the increase in serotype 6C in IPD among adults has so far remained without a concurrent increase in IPD in children.

Serotype 6D appeared to be extremely rare and was found among adult isolates only. All four isolates were from the post-PCV7-vaccination period, and two were post-PCV10/13. Serotype 6D isolated from invasive pneumococcal disease was first reported from Poland [22], where isolates were found both among children and adults. Interestingly, two of these isolates were ST948, an ST found in one of the German isolates as well. A further invasive 6D isolate was reported from Finland, having ST5163 [23]. In South Korea, 6D was found in four invasive isolates from children (all ST3171) and in one isolate from an adult patient (ST189) [24].

The INDEL was found in 46% of a subset of 95 6B isolates but not among 47 tested 6A isolates. This differs from what was found by Mavroidi et al. [7], who found INDEL frequencies of 20% in 6B and 2% in 6A.

The highest level of antibiotic resistance was found in serotype 6B isolates, followed by serotype 6A isolates, from both children and adults. This was in accordance with reports for the pre-vaccination period in many countries including the US, UK and Spain [25]–[27]. Serotype 6C only showed antibiotic resistance among adult isolates. One third of the resistant 6C isolates were multi drug resistant. Similar findings about serotype 6C antibiotic resistance were reported from Spain [28] and the US [29].

Serotype 6C isolates were found in connection with different forms of IPD, including pneumonia, sepsis and meningitis. This shows that serotype 6C is capable of causing a spectrum of IPD, comparable to other members of serogroup 6, as was previously reported [22]–[24].

The MLSTs found for the serotype 6C isolates in this study are, for the larger part, common to sequence types found among serogroup 6 isolates. Three of the four serotype 6D isolates belonged to clonal complexes common to serogroup 6 isolates. One 6D isolate (ST 8442) was not represented in the MLST database and remained a singleton in the eBURST analysis.

Two serogroup 6 isolates could not be typed correctly as 6A/6C or 6B/6D, respectively, using both factor sera as well as PCR analysis. However, both isolates have MLSTs that are common in serogroup 6. These isolates might represent as yet unknown serogroup 6 subtypes, and are under further investigation.

In Germany the prevalence of serotype 6A and 6B in IPD both among children and adults has diminished since the introduction of childhood conjugate vaccination. However, serotype 6C and possibly 6D are increasingly observed among IPD in adults. Future surveillance will reveal whether a cross-protection effect from higher-valent pneumococcal vaccination will reduce the prevalence of these serotypes.
